# Beneficial Impact of Vaccination Against SARS-CoV-2 on the Mental Health of IPF Patients

**DOI:** 10.3390/arm92060042

**Published:** 2024-11-06

**Authors:** Ioannis Tomos, Andriana I. Papaioannou, Zoe I. Daniil, Ilias E. Dimeas, Paraskevi Kirgou, Athena Gogali, Konstantinos Tatsis, Ilias Papanikolaou, Vasilios Tzilas, Argyrios Tzouvelekis, Panayiota Tsiri, Paschalis Steiropoulos, Pachalis Ntolios, Areti Xyfteri, Katerina Antoniou, Emmanouil Symvoulakis, Aggeliki Haritou, Maria Maniati, Lykourgos Kolilekas, Elvira-Markella Antonogiannaki, Vasiliki Apollonatou, Maria Kallieri, Kostas Samaras, Stylianos Loukides, Anna Karakatsani, Demosthenes Bouros, Effrosyni Manali, Spyros Papiris

**Affiliations:** 1 5th Pulmonary Medicine Department, SOTIRIA Thoracic Diseases Hospital of Athens, 11527 Athens, Greece; 2 2nd Pulmonary Medicine Department, General University Hospital “Attikon”, Medical School, National and Kapodistrian University of Athens, 12462 Athens, Greece; tzilasvasilios@gmail.com (V.T.); kantonogiannaki@gmail.com (E.-M.A.); vicky_apoll@hotmail.com (V.A.); mkallieri@yahoo.gr (M.K.); loukstel@med.uoa.gr (S.L.); akarakats@med.uoa.gr (A.K.); fmanali@otenet.gr (E.M.); papiris@otenet.gr (S.P.); 3 First Academic Department of Pneumonology, SOTIRIA Thoracic Diseases Hospital of Athens, Medical School, National and Kapodistrian University of Athens, 11527 Athens, Greece; papaioannouandriana@gmail.com; 4 Department of Respiratory Medicine, Medical School, University of Thessaly, 41110 Larissa, Greece; zdaniil@med.uth.gr (Z.I.D.); dimel13@hotmail.com (I.E.D.); paraskevi.kirgou@gmail.com (P.K.); 5 Department of Pneumonology, Medical School, University of Ioannina, 45500 Ioannina, Greece; athenagogali@yahoo.com (A.G.); konstantatsis@gmail.com (K.T.); 6 Pulmonary Department, Corfu General Hospital, 49100 Corfu, Greece; icpapanikolaou@hotmail.com; 7 Department of Respiratory Medicine, General Hospital of Patras, University of Patras, 26500 Patras, Greece; atzouvelekis@yahoo.gr (A.T.); tsiripanayiota@gmail.com (P.T.); 8 Department of Pneumonology, Medical School, Democritus University of Thrace, University General Hospital of Alexandroupolis, 68100 Alexandroupolis, Greece; steiropoulos@yahoo.com (P.S.); pascnt@hotmail.com (P.N.); 9 Private Practice, 24200 Messini, Greece; aretixyfteri@gmail.com; 10 Department of Thoracic Medicine and Laboratory of Molecular and Cellular Pneumonology, Medical School, University of Crete, 71500 Heraklion, Greece; kantoniou@med.uoc.gr; 11 Clinic of Social and Family Medicine, Department of Social Medicine, School of Medicine, University of Crete, 71500 Heraklion, Greece; symvouman@yahoo.com; 12 Private Practice, 45500 Ioannina, Greece; achdm2@gmail.com (A.H.); mar64man@yahoo.com (M.M.); 13 7th Pulmonary Department, SOTIRIA Thoracic Diseases Hospital of Athens, 15772 Athens, Greece; lykol@yahoo.gr; 14 Private Practice, 11527 Athens, Greece; kjsam74@gmail.com; 15 Iatriko Medical Center and National and Kapodstrian University of Athens, 15125 Athens, Greece; debouros@gmail.com

**Keywords:** mental health, depression, anxiety, Idiopathic pulmonary fibrosis, vaccination against COVID-19, vaccines, COVID-19

## Abstract

**Highlights:**

During the recent pandemic, IPF patients experienced an additional weight of stress related to their higher risk of being affected by severe COVID-19, the fear of forthcoming death, and the feeling of social isolation, especially during the initial phases of early lockdown implementation. Our study prospectively assessed the impact of vaccination against COVID-19 on mental health of IPF patients and revealed a significant decrease in anxiety and depression scores one month after the first dose of vaccines in IPF patients. It seems that vaccination offered an additional beneficial effect on the mental health of IPF patients by alleviating the perception of depression and anxiety.

**What are the main findings?**

- A higher Hospital Anxiety and Depression Scale score was detected before vaccination against COVID-19.
- A significant decrease in both anxiety and depression scores one month after the first dose of vaccines in IPF patients was found.

**What is the implication of the main finding?**

- It seems that vaccination also offered an additional beneficial effect on depression and anxiety in IPF patients.
- Vaccination against SARS-COV-2 offered at least a beneficial effect on the inception of patients’ mental health, an important issue in patients’ well-being and quality of life, in the course of IPF.

**Abstract:**

**Background**: Depression and anxiety represent significant comorbidities in idiopathic pulmonary fibrosis (IPF) patients, affecting their quality of life. The COVID-19 pandemic has had an uneven impact on global mental health. The Hospital Anxiety and Depression Scale (HADS) constitutes a validated tool to identify anxiety disorders and depression. The aim of this multicentre study was to evaluate the effect of COVID-19 vaccination on depression and anxiety in IPF patients. **Methods**: Consecutive IPF patients (median 73.5 years) who are regularly followed-up with were included in the study. Demographics, functional, and clinical were recorded. The HADS score was calculated before and one month after vaccination against COVID-19 in all participants. A Wilcoxon signed ranks test was conducted. **Results**: A total of 180 IPF patients (median 73.5 years) were included in the study. Among them, 145 patients (81%) received antifibrotic treatment. A significant reduction in HADS, both in anxiety and depression scales, was observed one month after vaccination against SARS-COV-2), independent of age, smoking, lung function impairment, and prior history of depression (*p* < 0.01). **Conclusions**: A higher Hospital Anxiety and Depression Scale score was detected before vaccination against COVID-19. It seems that vaccination also offered a beneficial effect on depression and anxiety in IPF patients, independent of age, smoking, lung function impairment, and prior history of depression.

## 1. Introduction

Usual Interstitial Pneumonia (UIP) and Idiopathic Pulmonary Fibrosis (IPF) constitute two irreversibly progressive and fatal fibrotic Interstitial Lung Diseases (ILDs), despite current treatment [1]. The recent coronavirus disease (COVID-19) pandemic imposed an additional health burden on exposed IPF patients, which are the most vulnerable population and are shown to experience an increased risk of severe acute respiratory syndrome (SARS) related to COVID-19 and high rates of hospitalisation and mortality, reaching approximately 50% [2,3]. Due to the above-reported dismal prognosis, the adoption of preventive measures including the imposition of the lockdown and vaccination, when it became available, was strongly advised to the IPF population, and patients were thankfully granted priority access to vaccination [4].

Among IPF patients, depression and anxiety are prevalent comorbidities affecting their quality of life [5,6]. During the course of the disease, the development of symptoms like dyspnoea is known to be related to the presence of depression in patients with IPF, revealing the importance of detecting this treatable comorbidity [7]. Moreover, it is generally accepted that when patients experience irreversible or unpredictable conditions, like that of the COVID-19 pandemic, depression and anxiety tend to become more severe. In general, the feeling of social isolation and the fear of forthcoming death represent frequent causes of psychological distress. A validated tool to evaluate accurately depression and anxiety in IPF is the Hospital Anxiety and Depression (HAD) score [7]. It constitutes a self-assessment questionnaire comprising seven questions for anxiety and seven for depression, and is a reliable scale to detect and evaluate the severity of both emotional disorders in the setting of a hospital outpatient clinic [8,9,10]. In the course of the pandemic and as soon as vaccines became available, the positive impact of vaccination on the mental health of vulnerable groups, especially those that faced the highest risk of hospitalisation or death, has been recognized [11].

We hypothesised that vaccination against SARS-COV-2, in addition to its preventative and protective role in IPF patients, may have also a beneficial impact on depression and anxiety in this group of patients. The aim of this multicentre, prospective study was to assess the potential impact of vaccination against COVID-19 on the mental health of IPF patients by evaluating the HAD score before and one month after vaccination.

## 2. Materials and Methods

The study was conducted between February 2021 and August 2021 and included IPF patients referred to 13 specialised ILD centres in Greece and were regularly followed-up with. In our country, vaccination for IPF patients was available after March 2021 [12]. Disease diagnosis was made according to the American Thoracic Society/European Respiratory Society/Japanese Respiratory Society/Latin American Thoracic Association consensus criteria for definite/probable UIP/IPF and after applying a multidisciplinary approach for each case. The study protocol was approved by the local Ethics Committee (ΠΝΕΥ, ΕΒΔ 104/17-2-2021) and participants provided written informed consent.

Demographic, epidemiological, functional, clinical, and microbiology data were collected for all participants. In addition, vaccination history, time since first diagnosis, comorbidities, and data on any immunosuppressive and/or antifibrotic treatment were recorded in detail. HAD score was assessed in all participants prior to vaccination against SARS-CoV-2 and one month after the first dose. Data were analysed using SPSS 18.0 for Windows (SPSS Inc, Chicago, IL, USA). Wilcoxon signed paired rank analysis was performed. A *p*-value <0.05 was considered statistically significant.

## 3. Results

**Figure 1:**
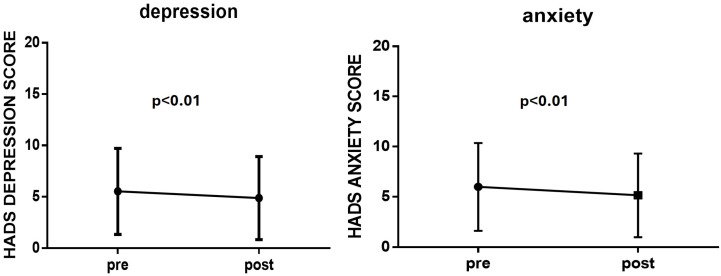
Significant reduction in HAD score in IPF patients one month after the first vaccination against SARS-CoV-2 (*p* < 0.01) registering an additional beneficial effect of vaccination on patients’ mental health.

In total, 180 IPF patients were included in the study [median age (IQR) 73.5 (68–78) years] with a male preponderance (78.9%), a median (IQR) forced vital capacity (FVC) of 79% (64–91%), and a diffusing capacity of the lungs for carbon monoxide (DLCO) at 46% (37–58%). The median disease duration was approximately 36 (20–53) months. The demographic and clinical characteristics of the study subjects are presented in Table 1. The majority of them had a history of smoking, while 81.1% received antifibrotic treatment, either nintedanib or pirferidone. During the study period that was at the beginning of the pandemic, only 2.8% of the participants suffered from SARS-COV-2 infection. Almost all patients were immunised against influenza (98.3%) and *Streptococcus pneumoniae* (97.8%), while 98.3% of them strongly intended to become vaccinated against SARS-CoV-2 prior to the circulation of vaccines. Arterial hypertension was the most common comorbidity among IPF patients [100 patients out of 180 (55.6%)], followed by diabetes mellitus (27.8%) and coronary artery disease (16.1%). Regarding the HAD score, the median (IQR) values for anxiety and depression in our IPF patients were low, 6 (2–9) and 5 (2–8), respectively, and a score less than 8 is considered a value indicative of the presence of anxiety disorders and depression. However, as shown in Figure 1, a higher Hospital Anxiety and Depression Scale score was detected before vaccination against COVID-19. Particularly, a significant reduction in the HAD score, both in depression and anxiety scale, one month after the first vaccination against SARS-CoV-2 (*p* < 0.01) (Figure 1) was observed, registering an additional beneficial effect of vaccination on IPF patients’ mental health and quality of life.

## 4. Discussion

During the recent pandemic, IPF patients experienced an additional weight of stress related to their higher risk of being affected by severe COVID-19, the fear of forthcoming death, and the feeling of social isolation, especially during the initial phases of early lockdown implementation, with all of them being frequent and well-known factors of psychological distress. Previous studies have reported increased levels of psychological distress and anxiety, in the general population since the onset of the pandemic [11,13,14]. Koltai J and co-workers, in a representative cohort of adults in the U.S., showed that vaccination against COVID-19 was also associated with a reduction in distress scores and alleviation of the perception of death [13]. Other observational studies confirm that unvaccinated people experience a high perceived risk of COVID-19 infection [15] and that fully vaccinated participants seem to present lower levels of anxiety and depression, providing further evidence that vaccination against SARS-CoV-2 may alleviate mental health symptoms [11].

Our study, for the first time, to the best of our knowledge, prospectively assessed the impact of vaccination against COVID-19 on the mental health of IPF patients, showing a significant decrease in anxiety and depression scores one month after the first dose of vaccines in IPF patients. Regarding the potential mechanism of this effect, we speculate that, as vaccination reduces the risk of severe infection, it may provide individuals with an additional sense of relief, a finding also observed in a previous study in a nationally representative cohort of U.S. adults [13].

In our cohort, the decrease detected in both HAD scores, although statistically significant, was particularly 1 unit in the medium of anxiety scale and 1 unit in the upper quartile range of the depression scale one month after the first vaccination against SARS-CoV-2. This is less than the minimal clinically significant difference necessary to reach a clinically meaningful improvement in patients’ symptom state, which is considered a limitation of the study. In addition, one month following vaccination may not be enough time to identify long-lasting changes, which might restrict the capacity to make firm conclusions on long-term impacts on mental health. One could speculate also that the reported clinical effects were quite mild and were not sufficient to significantly affect patients’ quality of life despite the statistical significance of the data. Nevertheless, this significant decrease in the score reveals at least a beneficial effect on the inception of patients’ mental health, an important issue in patients’ well-being and quality of life, in the course of this progressive and fatal disease. Further studies with additional measures to evaluate quality of life and overall well-being might offer further insight and a more thorough explanation of the therapeutic relevance of these findings. In this direction, even though the HAD score has been validated, using it alone cannot adequately represent the complexity of mental health issues; for instance, the HAM-A and HAM-D scales might be more helpful in future studies. However, this study represents a multicentre, prospective study with well-defined IPF patients assessing for the first time the impact of vaccination on mental health, an issue that deserves further investigation in IPF patients.

## 5. Conclusions

This study reports prospectively a significant decrease in HAD score one month after the first vaccination against COVID-19 in IPF patients, suggesting that vaccination offered an additional beneficial effect on the mental health of IPF patients by alleviating the perception of depression and anxiety.

## Figures and Tables

**Table 1:** Clinical characteristics of IPF patients (n = 180).

	n = 180
Age (years), median (IQR)	73.5 (68–78)
Gender (men), n (%)	142 (78.9)
Smoking, n (%)	
Current	94 (52.2)
Ex	38 (21.1)
Disease duration (months), median (IQR)	36 (20–53)
FVC (%), median (IQR)	79 (64–91)
DLCO (%), median (IQR)	46 (37–58)
Anti-fibrotic treatment (%)	146 (81.1)
SARS-CoV-2 infection, n (%)	
Yes	5 (2.8)
No	175 (97.2)
HAD-Anxiety score before vaccination, median (IQR)	6 (2–9)
HAD-Depression score before vaccination, median (IQR)	5 (2–8)
Vaccination against influenza, n (%)	177 (98.3)
Vaccination against *Streptococcus pneumoniae*, *n (%)*	176 (97.8)
Intention towards vaccination against SARS-CoV-2, n (%)	177 (98.3)
**Comorbidities**	
Coronary Artery Disease, n (%)	29 (16.1)
Arterial hypertension, n (%)	100 (55.6)
Diabetes mellitus, n (%)	50 (27.8)
Cancer, n (%)	18 (10)
Depression, n (%)	23 (12.8)

## Data Availability

The data presented in this study are available on request from the corresponding author due to ethical and privacy reasons.
